# Supplementary material for: Magneto-Mechanical Enhancement of Elastic Moduli in Magnetoactive Elastomers with Anisotropic Microstructures
Source: Materials (Basel). 2022 Jan 15;15(2):645. doi: 10.3390/ma15020645 (PMC8780743; doi:10.3390/ma15020645)
Supplement: Supplementary file 1 [file materials-15-00645-s001.zip › Support_information/Support_Information.pdf]

Supporting Information

Magneto-mechanical enhancement of elastic moduli in magnetoactive elastomers

Sanket Chougale[1], Dirk Romeis[1], Marina Saphiannikova[1]

[1]Leibniz Intitute of Polymer Research, Dresden

Corresponding author: chougale@ipfdd.de

The magneto-induced deformations and the MR effect are shown as a function of the initial aspect ratio γ_0 for different values ϕ_p at constant $\phi = 0.3$. In figures (S1), (S2) and (S3) the shifting of maxima can be seen with respect to the parameter ϕ_p for MAEs with SCs and SDs microstructures. The parameter ϕ_p is varied from $\phi_p = \phi$ to $\phi_p = 0.4$. All figures are plotted for $\mid \vec{H}_0 \mid = 470$ kA/m, $G_m = 17$ kPa.

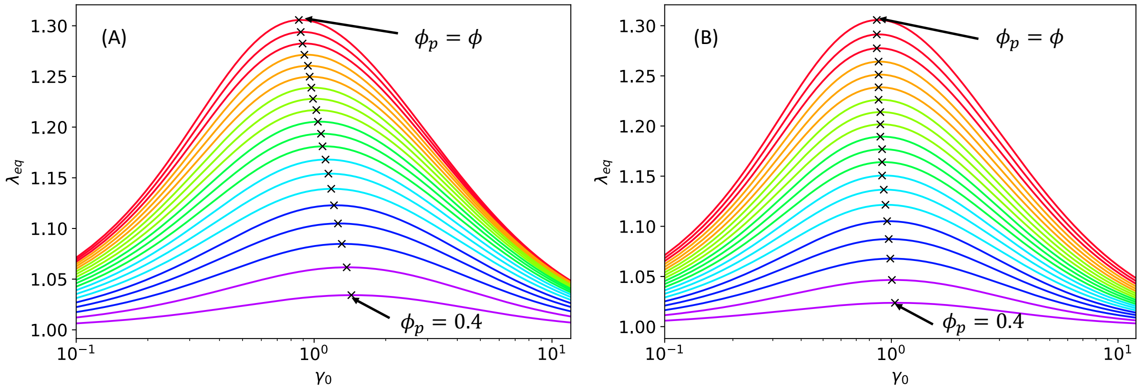

Figure S1: The magneto-induced elongations of anisotropic MAEs as a function of the initial aspect ratio γ_0 at different volume fractions ϕ_p and $\phi = 0.3$. (A) For smeared columns, (B) for smeared disks.

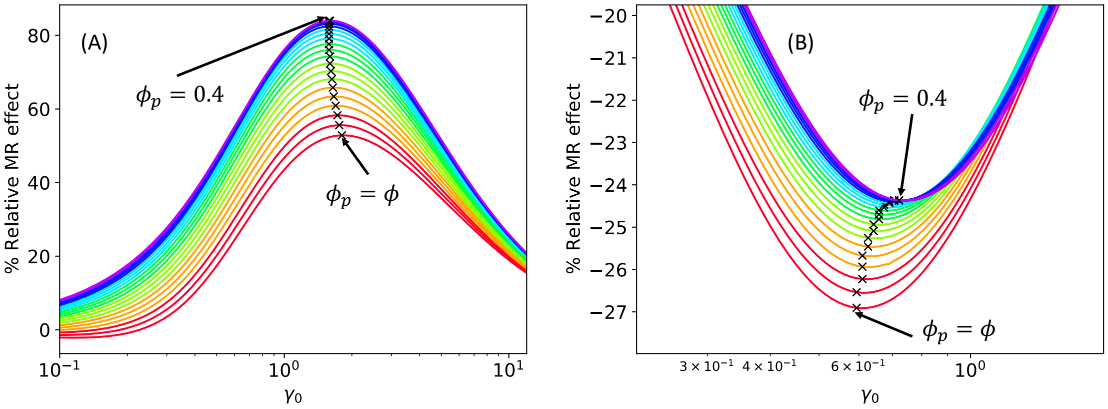

Figure S2: The magneto-rheological effect of anisotropic MAEs with SCs as a function of the initial aspect ratio γ_0 at different volume fractions ϕ_p and $\phi = 0.3$. (A) The MR effect along $\vec{H}_0$, (B) The MR effect perpendicular to $\vec{H}_0$.

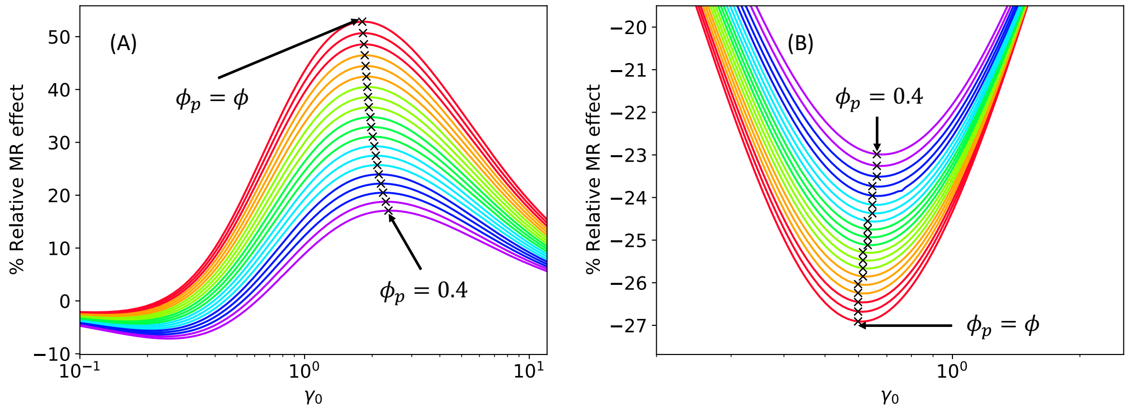

Figure S3: The magneto-rheological effect of anisotropic MAEs with SDs as a function of the initial aspect ratio γ_0 at different volume fractions ϕ_p and $\phi = 0.3$. (A) The MR effect along $\vec{H}_0$, (B) The MR effect perpendicular to $\vec{H}_0$.
