# Supplementary figures and images for: Magneto-Mechanical Enhancement of Elastic Moduli in Magnetoactive Elastomers with Anisotropic Microstructures
Source: Materials (Basel). 2022 Jan 15;15(2):645. doi: 10.3390/ma15020645 (PMC8780743; doi:10.3390/ma15020645)

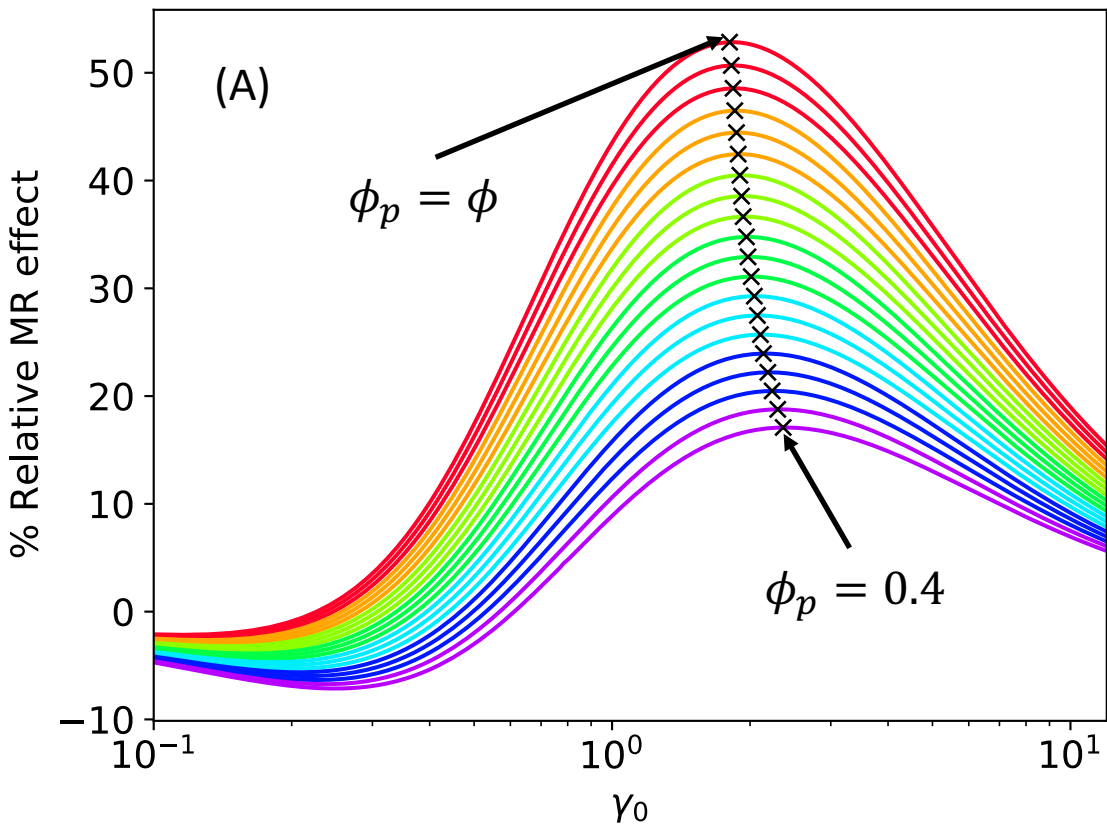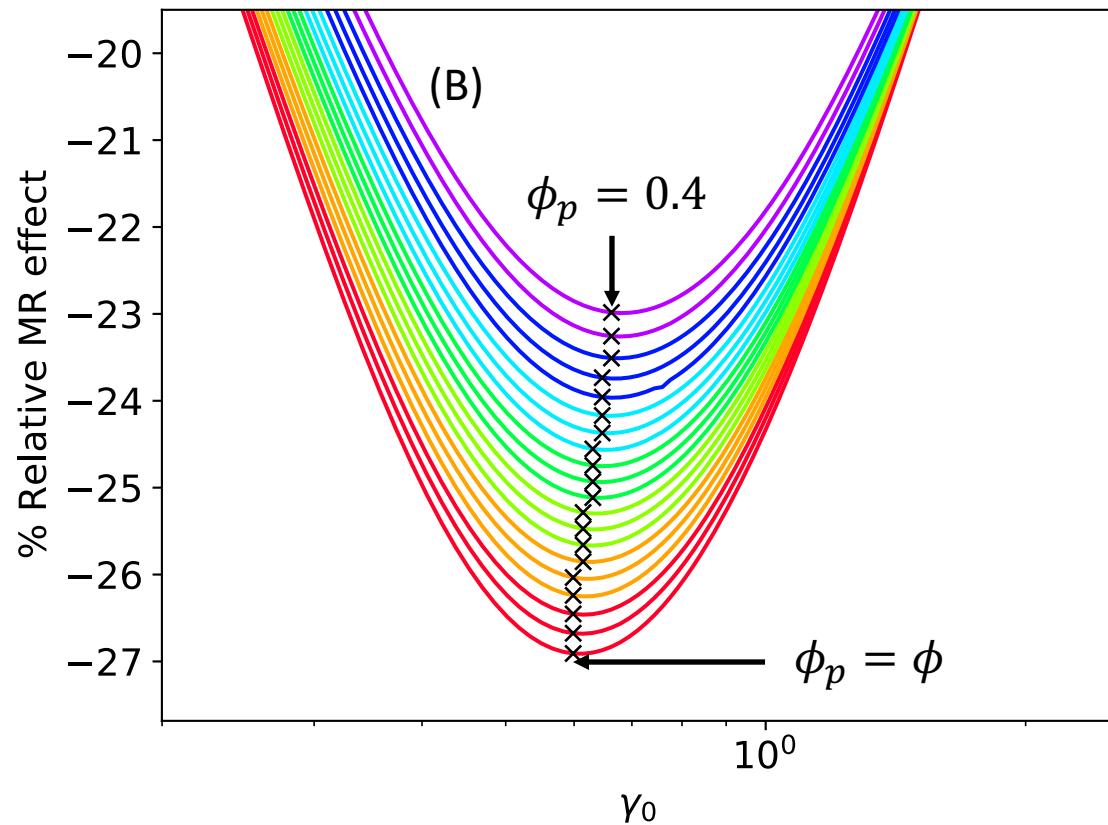

Supplement: Supplementary file 1 [file materials-15-00645-s001.zip › Support_information/S3_combine.pdf]

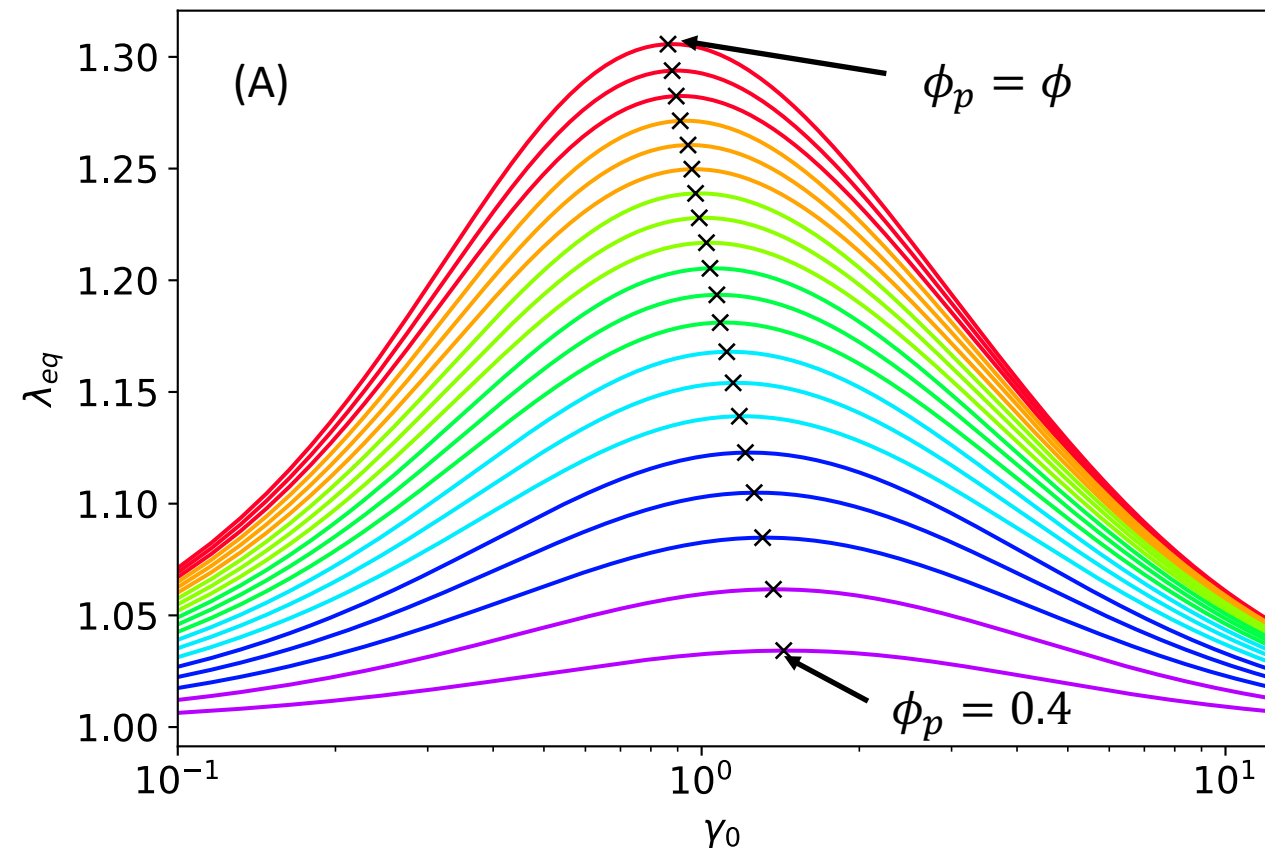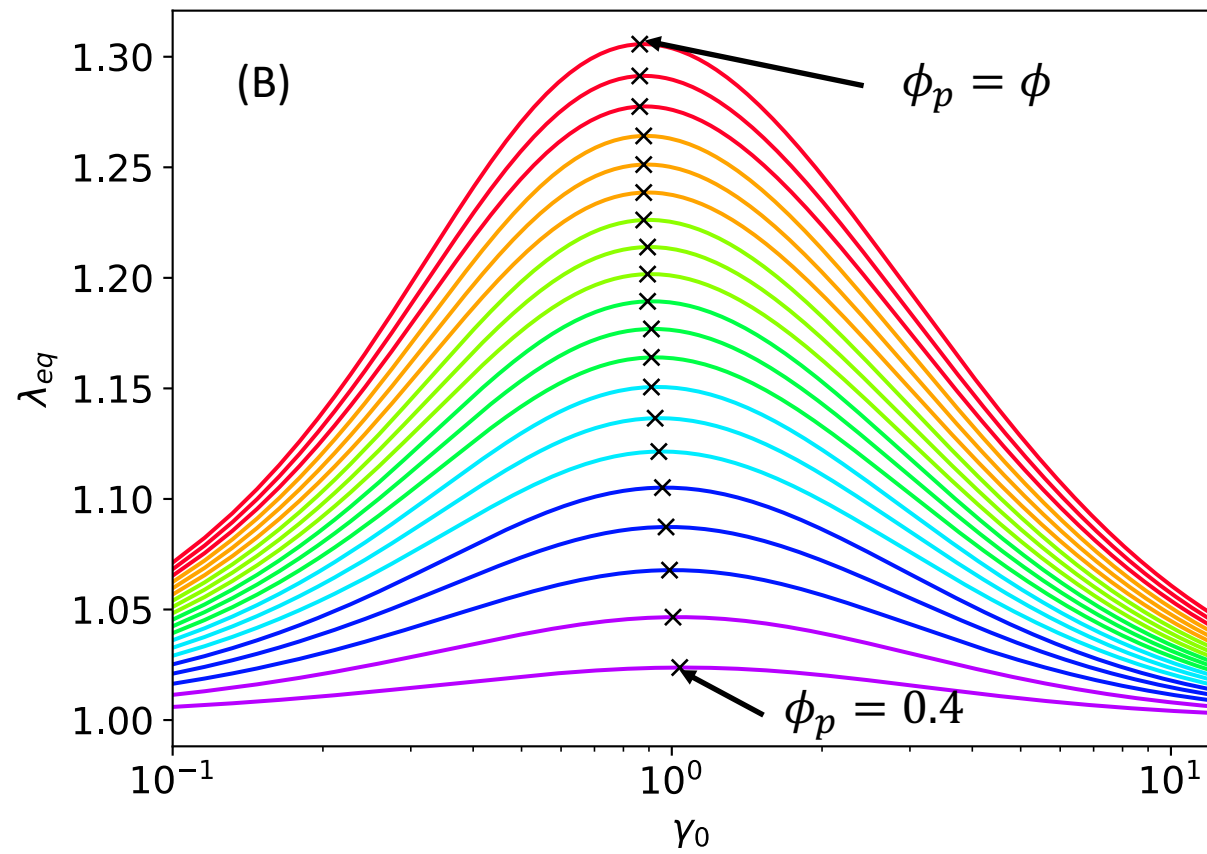

Supplement: Supplementary file 1 [file materials-15-00645-s001.zip › Support_information/S1_combine.pdf]

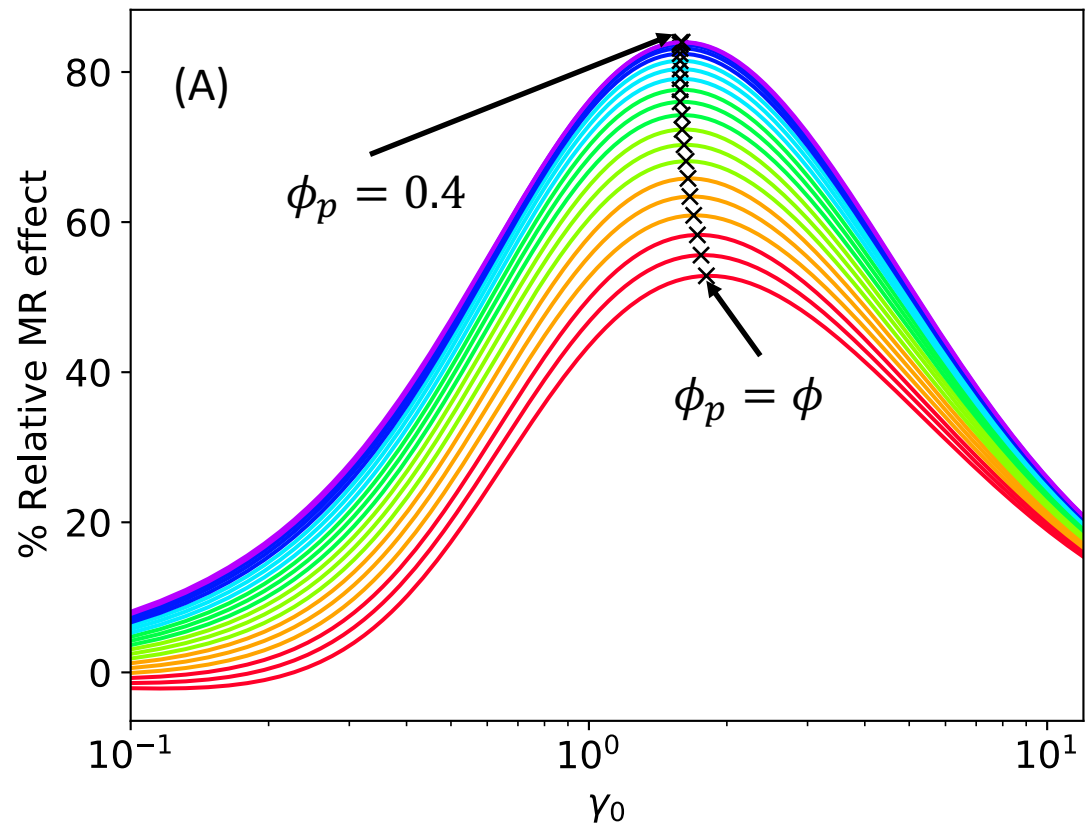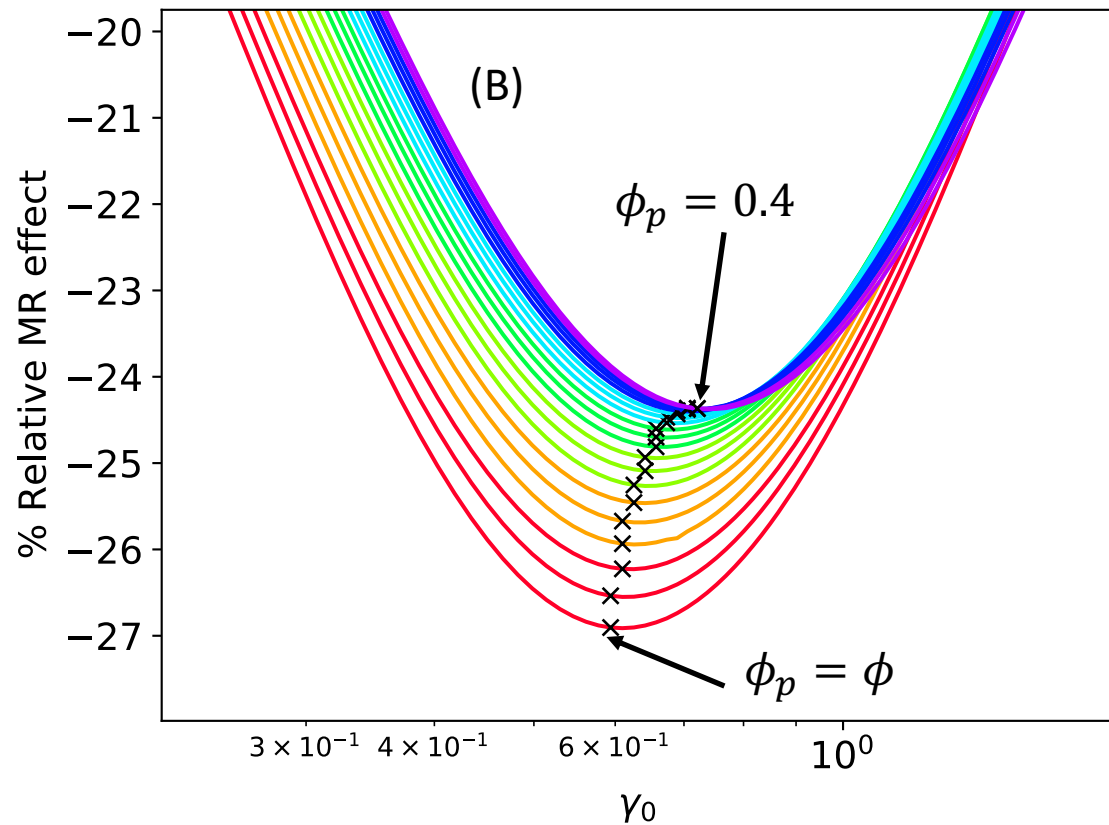

Supplement: Supplementary file 1 [file materials-15-00645-s001.zip › Support_information/S2_combine.pdf]
